# Characterization of sexual maturity-associated N6-methyladenosine in boar testes

**DOI:** 10.1186/s12864-024-10343-w

**Published:** 2024-05-07

**Authors:** Pengfei Zhang, Fei Zhang, Heming Sui, Xingyu Yang, Yiming Ji, Shenghao Zheng, Wei Li, Kun Cheng, Chonglong Wang, Jun Jiao, Xiaodong Zhang, Zubing Cao, Yunhai Zhang

**Affiliations:** 1 Anhui Province Key Laboratory of Local Livestock and Poultry, Genetical Resource Conservation and Breeding, College of Animal Science and Technology, Anhui Agricultural University, No.130 West Changjiang Road, Hefei, 230036 China; 2 National Animal Husbandry Service, Beijing, 100125 China; 3 Institute of Animal Husbandry and Veterinary Medicine, Anhui Academy of Agricultural Sciences, Hefei, 230031 China; 4 Anhui Haoyu Animal Husbandry Co., Ltd, Luan, 237451 China

**Keywords:** Pig, m6A, Testicular development, Fertility

## Abstract

**Background:**

The health and size of the testes are crucial for boar fertility. Testicular development is tightly regulated by epigenetics. N6-methyladenosine (m6A) modification is a prevalent internal modification on mRNA and plays an important role in development. The mRNA m6A methylation in boar testicular development still needs to be investigated.

**Results:**

Using the MeRIP-seq technique, we identify and profile m6A modification in boar testes between piglets and adults. The results showed 7783 distinct m6A peaks in piglets and 6590 distinct m6A peaks in adults, with 2,471 peaks shared between the two groups. Enrichment of GO and KEGG analysis reveal dynamic m6A methylation in various biological processes and signalling pathways. Meanwhile, we conjointly analyzed differentially methylated and expressed genes in boar testes before and after sexual maturity, and reproductive related genes (*TLE4, TSSK3, TSSK6, C11ORF94, PATZ1, PHLPP1* and *PAQR7*) were identified. Functional enrichment analysis showed that differential genes are associated with important biological functions, including regulation of growth and development, regulation of metabolic processes and protein catabolic processes.

**Conclusion:**

The results demonstrate that m6A methylation, differential expression and the related signalling pathways are crucial for boar testicular development. These results suggest a role for m6A modification in boar testicular development and provided a resource for future studies on m6A function in boar testicular development.

**Supplementary Information:**

The online version contains supplementary material available at 10.1186/s12864-024-10343-w.

## Background

N6-methyladenosine (m6A) modification is considered to be the most abundant internal post-transcriptional modification of RNA, especially in eukaryotic RNA [1, 2]. Writer/reader/eraser systems endow m6A modification with reversible characteristics and recognize a specific DRACH consensus motif where A is methylated D = A, G or U, R = A or G, and H = A, C or U [3]. m6A modification can occur on most types of RNA, including mRNA, tRNA, rRNA, snRNA, miRNA, lncRNA [4]. Much evidence suggests that m6A modification can regulate RNA stability, translation, export, structure and maturation of modified RNA [5, 6]. Single-cell sequencing data has demonstrated that RNA m6A regulators are expressed in almost all types of cells from the human testes [7]. Transcriptome-wide m6A-seq during porcine spermatogenesis indicated *SETDB1, FOXO1* and *FOXO3* are crucial for the determination of the fate of spermatogonial stem cells (SSCs) [8]. A *YTHDC2* mutation in mice resulted in male and female sterility due to impaired gametogenesis from four independent studies [9]. Loss of *YTHDC1* leads to extensively altered 3’ UTR length, resulting in massive alternative splicing defects in oocytes [10]. Low expression of FTO increases m6A levels, leading to mouse infertility [11].

The testicles play important roles in maintaining male characteristics, producing sperm, and secreting androgenic hormones. Testicles will undergo dramatic changes in male animals from fetus to adult. Normal testicular development is crucial for animal reproduction and sperm production. The adult testes contain germ cells and testis somatic cells, which include Sertoli cells, Leydig cells, and peritubular myoid cells. Testicular development and spermatogenic cell development is dependent on testis somatic cells [12]. The development of the testes largely determines the reproductive ability and health of males. In livestock breeding, the testicles are an important aspect of optimizing breeds. The study of testicular development molecular mechanisms is therefore of great significance for reproduction and breeding.

Studies have shown that testicular development is accompanied by various changes in epigenetic modifications, mRNAs and noncoding RNAs [13, 14]. In human, significant changes in DNMT expression and global DNA methylation levels in spermatogenic cells might contribute to development of male infertility in patients [15]. H3K4me2 plays important roles in spermatogenesis and cellular homeostasis, whereas H3K4me3 is implicated in nuclear architecture, RNA metabolism, spermatogenesis, and embryo development [16]. Studies have also demonstrated microRNAs mir-202-5p [17], mir-133B [18] and mir-202-3p [19] are critical for development of the Sertoli cell.

Epigenetic modifications play an important role in the development of the testes, and current m6A modifications are involved in the development of various tissues, including the testes. To further elucidate the role of m6A modifications, various sequencing methods have been developed. The first established high-throughput m6A sequencing method was MeRIP-seq based on m6A-specific antibodies [20]. UV-induced RNA-antibody crosslinking strategies have been adapted to produce m6A-CLIP and miCLIP by crosslinking immunoprecipitation to reveal the precise position of m6A [21, 22]. m6A-LAIC-seq was developed to reveal the census and complexity of the m6A epitranscriptome [23]. Based on detection of ionic current changes when a nucleic acid strand passes through a nanopore, nanopore sequencing was developed which can be performed on native nucleic acids, thus preserving nucleic acid modifications and enabling their direct detection [24]. Even if new sequencing methods for m6A continue to emerge, MeRIP-seq detection currently remains the common approach to profile m6A [25]. Moreover, MeRIP-seq has been applied to testes development [26, 27], embryo development [28], spermatogenesis [8] and stem cell differentiation [29].

Utilizing the mechanism of testicular development to accelerate the breeding process has been considered as an important strategy. Here, we used MeRIP-seq technology to characterize and investigate the differential expression of m6A on mRNAs in boar testes before and after sexual maturity. Our study will contribute to an m6A profile for boar testis development and identify key m6A modifications which could be used to improve boar reproductive performance in the future.

## Results

### Overview of m6A methylation and mRNA profiles in boar testes

**Fig. 1:**
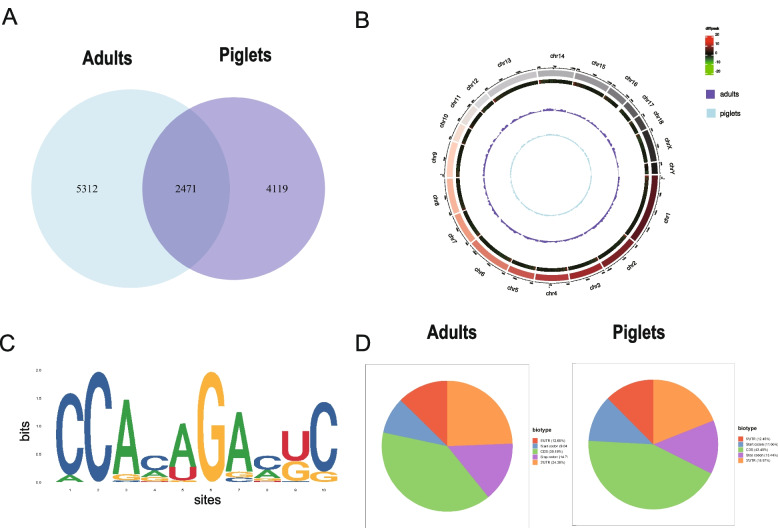
Overview of m6A methylation profiles in boar testes. **A** The number of common and specific m6A peaks in piglet and adult boar testes. **B** Distribution of m6A peaks across chromosomes in boar testes. **C** Top motifs with m6A peaks in boar testes. **D** Pie chart showing the peak in gene functional element region annotation (left piglets, right: adults)

The size, weight, and tissue sections of boar testes in piglets and adults were as previously described [30]. Using the MeRIP-seq technique, we identified 7,783 distinct m6A peaks in piglets and 6,590 distinct m6A peaks in adults, with 2,471 peaks shared between the two groups (Fig. 1A). Further, considering the m6A distribution on boar chromosomes, distribution analysis revealed that the m6A peaks differentially distributed in testes of piglets and adults (Fig. 1B). Furthermore, motif analysis results indicated that the two groups had the classic m6A DRACH consensus sequences (Fig. 1C). The majority of peaks were located at the start codon (9.04%VS11.66%), stop codon (14.79%VS13.44%), CDS region (39.18%VS43.48%), followed by the 3′UTR region (24.38%VS18.97%) and 5′UTR region (12.6%VS12.45%) (Fig. 1D).

### Functional analysis of differential m6A methylation of genes

**Fig. 2:**
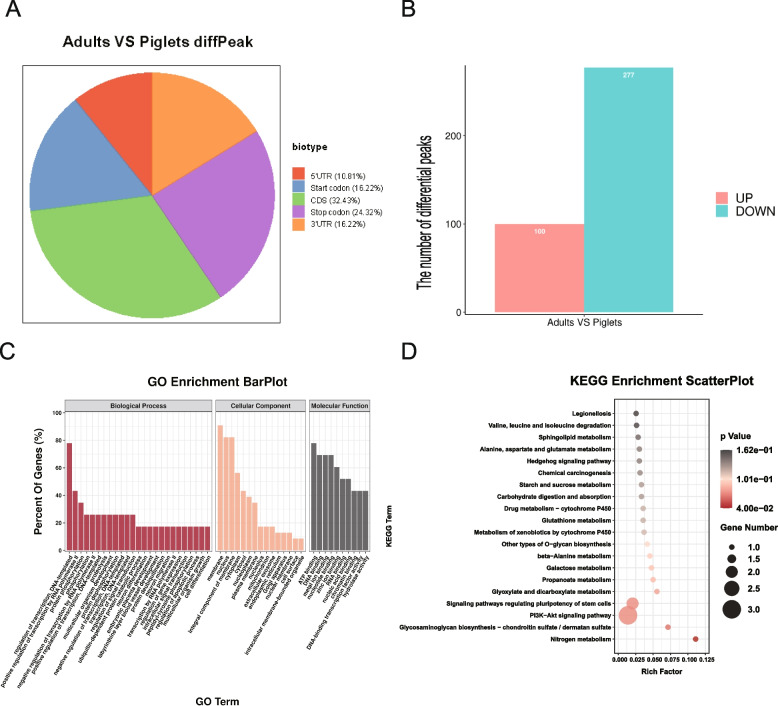
Functional analysis of differentially m6A methylated genes in boar testes before and after sexual maturity. **A** Distribution of differential methylation peaks on mRNA. **B** Statistical analysis of differential peaks. **C**, **D** GO (**C**) and KEGG (**D**) enrichment analysis of genes differentially expressed in piglets and adults

To characterize the potential function of the m6A modification in boar testes between piglets and adults, we compared differentially m6A methylated peaks in the two samples. Distribution analysis revealed that the differentially methylated peaks were also mainly concentrated in the start codon (16.22%), stop codon (24.32%), CDS region (32.43%), followed by the 3′UTR region (16.22%) and 5′UTR region (10.81%) (Fig. 2A). Further, compared to piglets, 100 m6A peaks were significant upregulated and 277 m6A peaks were significant downregulated in adults (Fig. 2B, Table S1). To elucidate the biological significance of the m6A peaks, GO and KEGG pathway analyses for differential methylation peaks were performed (Fig. 2C, D). GO annotation showed that m6A modifications on mRNAs were enriched in each of the molecular function, cellular component, and biological process modules. Their functions were mainly concentrated in aspects related to: 1) Molecular function: ATP binding, DNA binding, and RNA binding; 2) Cellular component: membrane, nucleus, and mitochondrion; 3) Biological process: protein phosphorylation, regulation of transcription, and cell differentiation (Fig. 2C). KEGG analysis showed that differential m6A modification was primarily associated with valine, leucine and isoleucine degradation, hedgehog signaling pathway and starch and sucrose metabolism (Fig. 2D).

### Functional analysis of differentially expressed genes

**Fig. 3:**
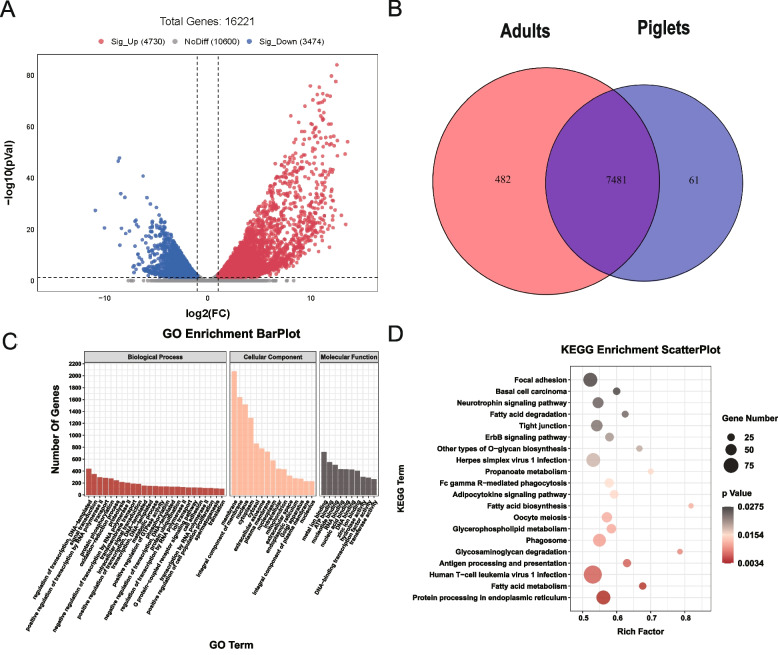
Functional analysis of differentially expressed genes in boar testes before and after sexual maturity. **A** Volcano plots showing the differentially expressed genes between the studied groups. **B** The number of common and specific genes in piglets and adult boar testes. **C**, **D** GO (**C**) and KEGG (**D**) enrichment analysis of genes differentially expressed in piglets and adults

To learn about changes in gene expression profiles in piglets and adults, using the RNA-seq technique, a total of 8,204 differentially expressed genes (DEGs) were detected between piglets and adults. We identified 4,730 up-regulated genes and 3,474 down regulated genes. A volcano plot showing the gene expression pattern of the DEGs is shown in Fig. 3A and described in detail in Table S2. Meanwhile, we identified 7,542 distinct genes in piglets and 7,963 distinct genes in adults, with 7,481 genes shared between the two groups (Fig. 3B). Enrichment analyses of GO terms and KEGG pathways were performed for differentially expressed genes. Enrichment analyses of GO terms showed results in the following processes: 1) Molecular function: ubiquitin-protein transferase activity, transforming growth factor beta binding, and phosphatidylinositol 3-kinase binding; 2) Cellular component: sperm principal piece, acrosomal vesicle, and male germ cell nucleus; 3) Biological process: spermatogenesis, spermatid development, and flagellated sperm motility (Fig. 3C). KEGG analysis indicated the differentially expressed genes had significant enrichment in pathways such as phosphatidylinositol signaling, the adipocytokine signaling pathway, glycerophospholipid metabolism and protein processing in endoplasmic reticulum (Fig. 3D).

### Joint analysis of differentially methylated and differentially expressed genes

**Fig. 4:**
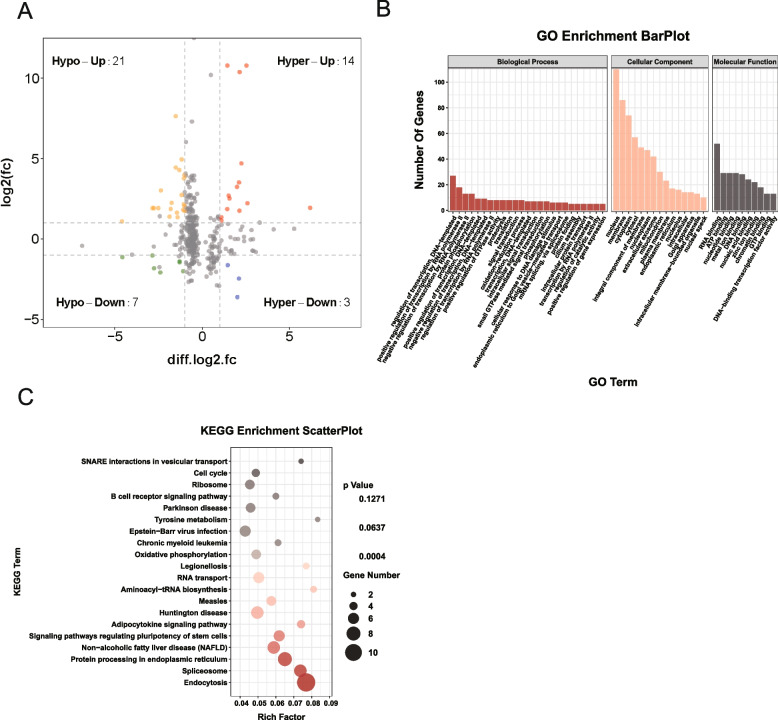
Joint analysis of differentially methylated and expressed genes in boar testes before and after sexual maturity. **A** Four quadrant plots showing differentially expressed genes with differentially methylated m6A peaks. **B**, **C** GO (**B**) and KEGG (**C**) enrichment analysis of the genes with a significant change in both m6A and mRNA levels

To further elucidate the differences between piglet and adult boar testes, we investigated the relationship between m6A methylation and RNA expression levels. By comparing m6A peaks and mRNA levels, four quadrant plots showed 14 mRNAs showing an up-regulation of m6A peaks and mRNA expression, 3 mRNAs showing an up-regulation of m^6^A peaks and down-regulation of mRNA expression, 21 mRNAs with down-regulation of m6A peaks and up-regulation of mRNA expression, and 7 mRNAs with down-regulation of both m6A peaks and mRNA expression (Fig. 4A, Table S3). Seven genes (*TLE4, TSSK3, TSSK6, C11ORF94, PATZ1, PHLPP1* and *PAQR7*) related to reproduction have been identified. Furthermore, GO and KEGG pathway enrichment analysis of the genes with a significant change in both m6A and mRNA levels is shown (Fig. 4B, C). GO enrichment results showed significant changes in genes related to proteolysis, signal transduction, phosphorylation, nucleus, membrane, cytosol, RNA binding, DNA binding and zinc ion while KEGG pathway analysis highlighted cell cycle, ribosome, the B cell receptor signaling pathway, spliceosome and endocytosis as being significant.

## Discussion

Testicular development is of great significance for livestock reproduction. Previous studies have demonstrated testicular development undergoes changes in epigenetic modifications and gene expression profiles. Accumulating studies have focused on the role of RNA m6A methylation in development and diseases. Studies have shown that writer/reader/eraser proteins are crucial for testicular development, oogenesis, spermatogenesis and m6A modification on mRNA leads to important changes in development of the reproductive system. Knockout of ALKBH5 has been shown to result in disorder of spermatogenesis and infertility in the male mouse [31]. Mettl3/Mettl14-mediated mRNA N6-Methyladenosine is known to modulate murine spermatogenesis [32]. In livestock, previous reports have shown that the dynamic role of m6A on RNA plays a key role in testicular development [26, 27, 33]. In our study, we selected the boar testis before and after sexual maturity to identify and profile the mRNA expression and m6A peaks using MeRIP-seq. Contrary to previous research on bovine testes [33], in our study, the m6A peak levels in the boar testicles decrease during development. However, in another study on bovine testes, a total of 2,351, 4,259, and 1,701 specific peaks were observed during prepuberty, puberty, and postpuberty stages, respectively [33]. According to a previous study on pig testes, 13,495, 10,552, and 11,824 methylated peaks were detected in D1, D75, and D150 groups, respectively [27]. Our data showed a decrease in m6A methylated peaks from D30 to D210. These results demonstrate that dynamic m6A methylation is playing a vital role in testicular development, with m6A fluctuating at different stages of testicular development. These results conclude the necessity of further research into m6A during testicular development. GO and KEGG pathway enrichment analysis indicate m6A peak alteration from piglets to adults in aspects such as cell differentiation, metabolism and the PI3K-Akt signaling pathway. These changes are related to the development of various cells in the testes.

Next, we jointly analyzed differentially methylated and expressed genes in boar testes, and reproduction-related genes (*TLE4, TSSK3, TSSK6, C11ORF94, PATZ1, PHLPP1* and *PAQR7*) were identified. TLE4 inhibits the dissociation of the CBF-1/RBP co-suppression complex and the expression of downstream transcription factors following downregulation of Notch1 during further differentiation of primordial germ cells (PGCs) [34]. The testis-specific serine kinase (TSSK) protein family members are widely expressed in testis and are involved in development of the testes [35]. TSSK3 is crucial for phosphorylation of multiple infertility-related proteins and plays an essential role in spermiogenesis [36, 37]. *TSSK6* (*SSTK*)-null mice were found to be infertile due to failure of sperm to relocate Izumo during the acrosome reaction [38]. C11ORF94 plays a critical role in sperm–egg interaction by controlling Izumo1 complex assembly [39, 40]. The *PATZ1* gene also has a critical role in spermatogenesis [41]. PHLPP1 regulates the NRNI activity of BRAP2 to influence spermatogenesis [42]. PAQR7 is an intermediary for progesterone to stimulate human sperm motility through a mechanism involving G protein activation [43]. GO and KEGG pathway enrichment analysis focused on cell cycle, ribosome, spliceosome and RNA transport. In summary, our results provide insights into m6A modification-regulated boar testicular development.

However, the development of the testes goes through a long-term process and various different stages [44]. Here, we only selected two representative stages for our studies. This may result in potential undetected changes in m6A at other developmental stages. In our study, we identify and profile the mRNA m6A peak only, but miRNAs and lncRNAs are also known to be crucial for testis development [45]. We recognize that we are probably missing information on m6A modification of miRNA and lncRNA during boar testis development in this study. The m6A modification of mRNA in whole boar testes tissue was measured before and after sexual maturity in the present study. However, the testes contain different cell types, and so we cannot distinguish in which cell type the m6A modification occurs. This hinders us from further understanding the role of m6A in boar testicular development. We hope to further specify the effect of methylation in boar testicular development in future studies.

## Conclusions

The results show that m6A methylation modifications are abundantly and dynamically expressed and may have important roles in boar testicular development. Thus, this study will provide a preliminary m6A profile and contribute to finding potential molecular markers for boar testicular development.

## Materials and methods

### Animals and tissue collection

These experiments were performed as previously described [30]. Briefly, Landrace boar piglets (30-day-old, three boars) and adult pigs (210-day-old, three boars) were obtained from Anhui Hoshine Agro-Pastoral Co., Ltd., Anhui, China and slaughtered after electric shock stunning. The testicular skin was removed. The testicular samples were immediately cut into small pieces, transferred into cryogenic vials, and stored in liquid nitrogen for subsequent library construction and sequencing.

### RNA isolation, library construction and sequencing

According to the manufacturer’s instructions (Invitrogen, Carlsbad, CA, USA), total RNA was isolated from testicular samples using the Trizol method. Briefly, testicular samples were ground in a low-temperature environment, and total RNA was isolated using TRIzol ™ Reagent. The isolated RNA was treated with DNase I to remove genomic DNA contamination. The total RNA quality and quantity were analyzed on a Bioanalyzer 2100 and RNA 6000 Nano LabChip Kit (Agilent, CA, USA). Samples with RIN number > 7.0 were deemed suitable for further analyses.

In order to separate poly (A) RNA, according to the manufacturer’s (Invitrogen, USA) instructions, oligomeric (dT) coupled magnetic beads were used for two rounds of purification of the total RNA. Purified poly(A) mRNA fractions were fragmented into ~ 100-nt-long oligonucleotides using divalent cations under elevated temperature. Subsequently, poly (A) mRNA fragments were subjected to incubation with m6A-specific antibody (No. 202003, Synaptic Systems, Germany) in an IP buffer (50 mM Tris–HCl, 750 mM NaCl and 0.5% Igepal CA-630, 0.5 μg /μl BSA) for 2 h at 4℃, and allowed to incubate with protein-A beads. The mixture was then washed with IP buffer three times and m6A-positive RNA was eluted with elution buffer (1 × IP buffer and 6.7 mM m6A). Eluted RNA was precipitated by 75% ethanol. Eluted m6A-containing fragments (IP) and untreated input control fragments are converted to final cDNA library in accordance with a strand-specific library preparation by the dUTP method. The average insert size for the paired-end libraries was ~ 100 ± 50 bp. We then performed the paired-end 2 × 150 bp sequencing on an Illumina Novaseq™ 6000 platform at LC-BIO Bio-tech ltd (Hangzhou, China) following the vendor’s recommended protocol.

### Bioinformatics analysis of m6A-seq and RNA-seq data

Firstly, CutAdapt (http://pypi.python.org/pypi/cutadapt) software were used to remove the reads that contained adaptor contamination, low quality bases and undetermined bases to obtain clean data. Then sequence quality of IP and input of all samples was verified using fastp software [46]. Subsequently, the high-quality clean reads were mapped to the to the genome of Sus scrofa (Version: sus_scrofa_ensembl_V88) with HISAT2 [47]. Mapped reads of IP and input libraries were provided for the R package, exomePeak [48], which identifies m6A peaks with.bed or.bam format files that can be adapted for visualization on the UCSC genome browser or IGV software (http://www.igv.org/). MEME [49] and HOMER [50] were used for de novo and known motif finding followed by localization of the motif with respect to peak summit using in-house perl scripts. Called peaks were annotated by intersection with gene architecture using ChIPseeker [51]. StringTie [52] was then used to measure expression levels for all mRNAs from input libraries by calculating FPKM (FPKM = [total exon_fragments/mapped_reads (millions) × exon_length (kB)]). The differentially expressed mRNAs were selected with log2 (fold change) > 1 or log2 (fold change) < -1 and *p-*value < 0.05 by R package edgeR [53].

### Gene Ontology (GO) and Kyoto Encyclopedia of Genes and Genomes (KEGG) pathway enrichment analysis

Differentially expressed genes were selected for Gene Ontology (GO) analysis and Kyoto Encyclopedia of Genes and Genomes (KEGG) analysis. Differential expression was classified into the three categories of the GO database: biological processes, cellular components, and molecular functions. The KEGG database was used to ascribe differential expression to biological mechanisms and cellular pathways. GO and KEGG enrichment analysis was carried out with online tools (http://geneontology.org and http://www.kegg.jp/kegg).

### Supplementary Information

**Supplementary Material 1.** 

**Supplementary Material 2.** 

**Supplementary Material 3. **

## Data Availability

The datasets generated for this study can be found in NCBI SRA under accession PRJNA1031007. The datasets contain MeRIP–seq data and RNA-seq data, with input in the file name representing RNA-seq data and with IP in the file name representing MeRIP–seq data. Other data presented in this study are available on request from the corresponding author.
